# Dislocability of Localization Devices for Nonpalpable Breast Lesions: Experimental Results

**DOI:** 10.1155/2014/425823

**Published:** 2014-03-06

**Authors:** David Kaul, Eva Fallenberg, Felix Diekmann, Volker Budach, Martin Maurer

**Affiliations:** ^1^Department of Radiation Oncology, Charité School of Medicine and University Hospital, Campus Virchow-Klinikum, Augustenburger Platz 1, 13353 Berlin, Germany; ^2^Department of Radiology, Charité School of Medicine and University Hospital, Campus Virchow-Klinikum, Augustenburger Platz 1, 13353 Berlin, Germany; ^3^Krankenhaus St. Joseph-Stift, Institut für Radiologie, Schwachhauser Heerstraße 54, 28209 Bremen, Germany

## Abstract

*Purpose*. For accurate resection of nonpalpable malignant breast lesions with a tumor-free resection rim, an exact and stable wire localization is essential. We tested the resistance towards traction force of different localization devices used in our clinic for breast lesions in two types of tissue. *Materials and Methods*. Eight different commercially available hook-wire devices were examined for resistance towards traction force using an analogue spring scale. *Results*. Most systems showed a high level of movement already under small traction force. Retractable systems with round hooks such as the Bard DuaLok , the Fil d'Ariane, and the RPLN Breast Localization Device withstood less traction force than the other systems. However, the Bard DuaLok system was very resistant towards a small traction force of 50 g when compared to the other systems. The Ultrawire Breast Localization Device withstood the most traction force in softer tissue and Kopans Breast Lesion Localization Needle withstood the most force in harder tissue. *Conclusion*. The Ultrawire Breast Localization Device and Kopans Breast Lesion Localization Needle withstood the most traction force. In general retractable systems withstand less traction force than nonretractable systems.

## 1. Introduction

With the introduction of worldwide breast cancer screening programs, an increasing number of nonpalpable breast lesions are detected in clinically asymptomatic patients [1]. Approximately 15–20% of such lesions are proved to be malignant and require surgical removal following radioguided localization of the lesion [2]. Modern imaging allows us to identify potentially malignant breast lesions even before they become palpable. Therefore reliable techniques are needed to identify these nonpalpable lesions during surgery. Wire-guided localization (WGL) is the most commonly used localization method [3]. Several localization devices with different hook-systems are commercially available. Most localization devices consist of a wire or thread with a hook system. These localization devices are inserted into the lesion under radiographic guidance via a needle system and should provide for sufficient hold of the hooks in the tissue.

Malignant tumors of the breast usually present as thick, hard nodules while the breast tissue itself is in most cases of softer consistency. In small lesions the hook can either be located within the tumor itself or next to it in the softer surrounding normal breast tissue. The thickness of breast tissue itself may also vary depending on age and history of pregnancy.

Since lesions are often not palpable, breast surgeons have to rely on the correct localization to remove the entire lesion. However, the protruding end of the localization wire from the breast marking procedure is at risk of displacement before or during surgery [4]. We probed the resistance of different hook systems against traction force to overcome this putative threat to the proper removal of malignant lesions.

## 2. Materials and Methods

Resistance capability towards traction force of eight commercially available hook-wire devices for breast lesion localization was examined in two types of tissue (*n* = 1) including Bard Ghiatas Beaded Breast Localization Wire with Stiffened Section (C. R. Bard, Inc., NJ, USA), Pluslok Brust Lokalisationskanüle (Peter Pflugbeil GmbH, Zorneding, Deutschland), Bard DuaLok Breast Localization Wire (C. R. Bard, Inc., NJ, USA), Ultrawire Breast Localization Device (INRAD Inc., MI, USA), Fil d'Ariane (Peter Pflugbeil, Zorneding, Germany), RPLN Breast Localization Needle with nitrol “J” wire (CP Medical Inc., OR, USA), X-Reidy Breast Lesion Localization Needle (Cook Medical Inc., IN, USA), and Kopans Breast Lesion Localization Needle (Cook Medical Inc., IN, USA).

**Figure 1:**
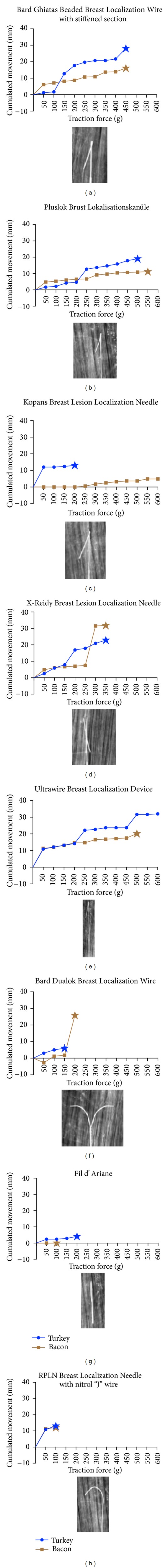
Cumulated movement of localization devices in different types of tissue after the application of traction force. A star at the end of a curve indicates the traction force with which the wire was completely removed from the tissue.

The hook system shapes fall into different groups (Figure 1): single-hook systems (Figures 1(a)–1(c), 1(e), 1(g), and 1(h)) and multiple-hook systems (Figures 1(d) and 1(f)) are found. In addition, shapes are built to withstand either pulling force alone (Figures 1(a)–1(c)) or to withstand both pulling and pushing forces (Figures 1(d), 1(e), and 1(f)–1(h)). In addition, some systems are designed to remain within the marked tissue (Figures 1(a)–1(e)), whereas retractable systems provide an opportunity to remove the hook noninvasively (Figures 1(f)–1(h)).

Two types of tissue were tested: as a substitute for softer human breast tissue turkey breast was used and as a thicker and harder tissue we used pork ham. We only probed the resistance capability towards pulling force with our experimental apparatus. Tissues were examined at room temperature. The tissues were compressed in the mammography unit (Senographe Essential, General Electric Healthcare, Chalfont St. Giles, UK) using a compression force of 10 N. Localization devices were inserted approx. 5 cm into the compressed tissues following the manufacturer's recommendations. Traction force was applied in each case for 1 second using an analogue Spring Scale (600 g Spring Scale with ISO 900 calibration, ATP GmbH, Ettenheim, Germany) starting with 50 g of pulling force (1000 g = 9.81 Newton). Pulling force was increased in steps of 50 g. A mammography image was taken after each time of force application to document a movement of the hook within the tissue. The extent of displacement of the hook-wire was measured using a measurement tool in the image viewing system (Centricity PACS, GE Medical Systems, WI, USA).

Statistics were performed using GraphPad Prism 6.02, GraphPad Software, Inc., CA, USA. Systems that withstood 600 g of pulling force were treated as having been pulled out at 650 g for statistical analysis. A *P* value of less than 0.05 was considered as statistically significant.

## 3. Results

None of the tested devices withstood a pulling force of 600 g in both tissue types tested. The Ultrawire Breast Localization Device (Figure 1(e)) withstood 600 g of pulling force in turkey breast, while Kopans Breast Lesion Localization Needle withstood 600 g of pulling force in bacon (Figure 1(c)). However, the Ultrawire Breast Localization Device (Figure 1(e)) was already moved more than 1 cm in both types of tissue after application of a traction force of only 50 g. Kopans Breast Lesion Localization Needle (Figure 1(c)) was moved more than 1 cm in turkey breast following a traction force of 50 g. Both Bard Ghiatas Beaded Breast Localization Wire (Figure 1(a)) and the Pluslok Brust Lokalisationskanüle (Figure 1(b)) withstood more than 400 g of traction force in both tissues tested. The best result among the retractable devices was obtained with the Bard DuaLok Breast Localization Wire, which was moved less than 5 mm after a traction force of 50 g.

Some devices did not withstand more than 200 g of pulling force in both tissue types. The Bard DuaLok Breast Localization Wire withstood 200 g of pulling force in bacon and 150 g in turkey; the Fil d'Ariane withstood 100 g of pulling force in bacon and 200 g in turkey breast. The RPLN Breast Localization Needle with nitrol “J” wire withstood 100 g of pulling force in bacon and 100 g in turkey breast. The Bard DuaLok Breast Localization Wire showed a negative movement after applying a force of 50 g (Figure 1(f)); this might be due to the tension provoked in the two wings of the device which led to a springing back of the hook in the tissue after 50 g of force was applied.

In general, retractable systems needed significantly less force to be pulled out of the tissue than nonretractable systems (*P* = 0,034 in turkey and *P* = 0,0016 in bacon, *t*-test). Retractable and nonretractable systems showed similar resistance towards low traction force of 50 g (*P* = 0,9538 in turkey and *P* = 0,5450 in bacon, *t*-test).

There was no significant difference between resistance towards traction force in nonretractable systems with single hooks and nonretractable systems with multiple hooks (*P* = 0,9252, *t*-test). Retractable multiple hook systems and retractable single hook systems did not show significant differences either (*P* = 0,2846, *t*-test). Systems that withstand pulling and pushing forces on the one hand and systems that withstand only pulling force on the other hand did not show significant differences (*P* = 0,0813, *t*-test).

There was no significant difference between the traction force required to pull out various devices from soft and hard types of tissue (*P* = 0,675, *t*-test).

## 4. Discussion

Accurate intraoperative localization of breast lesions can be a difficult task, with the potential to cause complications such as unnecessary large resection margins or, in the worst case, insufficient removal of breast lesions. Even though localization devices are widely accepted and used nowadays, reported dislocations [5, 6] raise some concern. Therefore, we tested various localization devices for breast lesions used in our clinic for resistance capacity towards traction force in two types of tissue. None of the tested devices withstood pulling force of 600 g in both tissue types. As expected, retractable systems with round hooks such as the Bard DuaLok, the Fil d'Ariane, and the RPLN Breast Localization Device withstood much less traction force compared with the other systems tested. This agrees with a previous study reporting a lower resistance capacity towards a pulling force in retractable systems [7]. However, we observed no significant difference in resistance towards small traction force in retractable and nonretractable systems. The retractable Bard DuaLok displayed the highest resistance towards a traction force of 50 g when compared with the other two retractable systems.

The Ultrawire Breast Localization Device withstood the highest traction force in turkey. However, the device was dislocated by more than one cm after application of 50 g of traction force in both tissue types. While the Kopans Breast Lesion Localization Needle withstood the highest force in bacon, part of the hook broke off when using turkey instead and remained stuck within the tissue. This is in agreement with the findings of Langen et al., who reported a breaking tendency under high traction force for systems with high resistance towards pulling force [7]. The resistance of 600 g measured for Kopans Breast Lesion Localization Needle in bacon is consistent with the results of Urrutia et al. reporting a maximum resistance of 780 g and Schoenberger et al. who found the maximum resistance to be 454 g [8, 9]. Determined resistance of 200 g for Kopans Breast Lesion Localization Needle in turkey is in accordance with earlier studies by Langen et al. giving a value of 204 g [7].

However, very high traction forces of more than 500 g are unlikely to be applied to a localization device *in situ*. Therefore, it is more important to focus on smaller traction forces. Here the Bard DuaLok system showed the best results.

Our study had some limitations. Firstly differences in breast compression could bias the results compared to other studies or everyday clinical life. Secondly inhomogeneities in the tissues might have compromised the results. Thirdly it has to be mentioned that we only tested the systems for pulling and not for pushing force, which can also occur in everyday clinical life.

## 5. Conclusion

The Ultrawire Breast Localization Device and Kopans Breast Lesion Localization Needle withstood the most traction force. In general retractable systems withstand less traction force than nonretractable systems. The fact that application of just a small traction force can already lead to displacement of more than 1 cm in several systems tested underlines the importance of proper fixation of the device.
